# Elevational sensitivity in an Asian ‘hotspot’: moth diversity across elevational gradients in tropical, sub-tropical and sub-alpine China

**DOI:** 10.1038/srep26513

**Published:** 2016-05-23

**Authors:** L. A. Ashton, A. Nakamura, C. J. Burwell, Y. Tang, M. Cao, T. Whitaker, Z. Sun, H. Huang, R. L. Kitching

**Affiliations:** 1 Environmental Futures Research Institute and Griffith School of Environment, Griffith University, Nathan, Queensland 4111, Australia; 2 Key Laboratory of Tropical Forest Ecology, Xishuangbanna Tropical Botanical Garden, Chinese Academy of Sciences, Mengla, Yunnan 666303, China; 3 Department of life sciences, Natural History Museum, London, SW7 5BD, UK; 4 Biodiversity Program, Queensland Museum, South Brisbane, Queensland 4101, Australia; 5 Crowtrees, Low Bentham, Lancaster, LA2 7EE, UK; 6 Lijiang Forest Ecosystem Research Station, Kunming Institute of Botany, Chinese Academy of Sciences, Kunming 650201, Yunnan, China

## Abstract

South-western China is widely acknowledged as a biodiversity ‘hotspot’: there are high levels of diversity and endemism, and many environments are under significant anthropogenic threats not least climate warming. Here, we explore diversity and compare response patterns of moth assemblages among three elevational gradients established within different climatic bioregions - tropical rain forest, sub-tropical evergreen broad-leaved forest and sub-alpine coniferous forest in Yunnan Province, China. We hypothesised that tropical assemblages would be more elevationally stratified than temperate assemblages, and tropical species would be more elevationally restricted than those in the temperate zone. Contrary to our hypothesis, the moth fauna was more sensitive to elevational differences within the temperate transect, followed by sub-tropical and tropical transects. Moths in the cooler and more seasonal temperate sub-alpine gradient showed stronger elevation-decay beta diversity patterns, and more species were restricted to particular elevational ranges. Our study suggests that moth assemblages are under threat from future climate change and sub-alpine rather than tropical faunas may be the most sensitive to climate change. These results improve our understanding of China’s biodiversity and can be used to monitor future changes to herbivore assemblages in a ‘hotspot’ of biodiversity.

First proposed by Mittermeier and Myers as long ago as 1988, the conservation ‘hotspot’ idea has gained considerable traction and has, in part, underpinned national and international conservation policy settings ever since^1^,^2^,^3^. An essential part of the ‘hotspot’ concept has been the idea of vulnerability and endangerment which, in the original formulation, was interpreted as vegetation transformation and the lack of large, formally protected areas^4^. Since the inception of the ‘hotspot’ idea, the threats represented by anthropogenic global warming and their likely impact on biodiversity have received increasing attention^5^.

The biological provinces of south-west China have featured as a ‘hotspot’ from the conception of this idea. Myers *et al*.^2^ pointed out that this region has lost over 90% of its original primary vegetation, yet is very species rich with over 12000 species of plants, of which more than 25% are endemic. In the extreme south of this region and beyond lies a second ‘hotspot’, Indo-Burma, which harbours over 15000 plant species, 400 mammal species and 1200 bird species^6^. The evolutionary and biogeographical reasons for this regional hyper-diversity are still a matter for debate. Certainly classical Palaearctic and Oriental faunas overlap here^7^,^8^ although this begs the questions of why this should lead to enhanced co-existence and diversity. Undoubtedly, the highly dissected and geologically heterogeneous nature of this mountainous landscape plus the general parallel north-south trending mountain ranges^9^ play a part in both engendering and maintaining this high diversity. The location of the region at the very core of mainland East Asia with large areas (before clearing) of habitat available at the latitude may also have acted to produce the very large species pool.

Climate change is having a marked effect on biodiversity, through shifts in elevational and latitudinal range, local extinctions, changes in species composition and phenology, as well as a suite of ecosystem-level impacts^10^. The impacts of climate change on biodiversity are difficult to measure, especially in areas such as south-west China where there is little historical information about distributions of taxa. Western Sichuan and the Yunnan-Guizhou plateaux have experienced a clear warming trend and a decline in rainfall days over the last 40 years of the 20^th^ century. Biodiversity reduction, ecosystem degradation and desertification have followed these changes^11^.

Elevational gradients may be one of the few ways to study the effects of climate change on ecological communities given a lack of historical data^12^,^13^. Forested elevational gradients representing sets of adjacent climates are excellent tools for such studies^12^,^14^,^15^,^16^,^17^,^18^, encompassing, in a small geographical area, a range of environmental factors that shift in a predictable way. For every 100 m of increased elevation, the temperature decreases by around 0.6 °C^19^. A suite of other environmental factors shift in concert, including soil chemical properties^20^ and precipitation, the latter often also influenced by the presence of a cloud cap^21^. The cloud cap results in additional water inputs through orographic precipitation, influencing a suite of environmental factors and resulting in modified community structure^22^. Increased temperatures are predicted to raise the average level of the cloud base^23^ and, given the high level of endemism in tropical cloud forest systems, this is an area of conservation concern^24^.

Tropical fauna, especially ectotherms, are considered to be thermal specialists^25^. Indeed some studies have shown that the thermal tolerance of tropical species may be narrower than species of the same taxonomic group found in temperate regions^26^,^27^. Tropical species generally experience more stable climates with fewer seasonal extremes, in contrast to their temperate counterparts which experience regular, often extreme, seasonal climatic variations. As a result, tropical species may reach and exceed their thermal maxima more readily than temperate species, resulting in them having more restricted distributions with smaller elevational ranges. These results and the deduced sensitivities of species to climate change are, however, primarily derived from laboratory experiments (to measure CT_max_ and CT_min_) investigating the impact of ambient temperature on individual species separately. Recent studies have suggested that changes in air temperature alone cannot be used to deduce vulnerability of organisms to climate change as other factors such as solar radiation, relative humidity, wind exposure and morphological (e.g. body colour), phenological (period during which species are active) and behavioural (e.g. ability to seek thermal refugia) characteristics of individual species all interplay to generate differential sensitivities to climate change under field conditions^28^,^29^,^30^. More investigation is therefore warranted to examine sensitivities of organisms under field conditions^31^.

Although plants and vertebrates initially received the lion’s share of attention in conservation assessments, increasing attention is now being paid to invertebrates, although this level of attention is yet to match their biotic preponderance in terrestrial ecosystems^32^. Terrestrial invertebrates are, on the one hand, key drivers of ecological processes^33^ and, on the other, excellent predictors of environmental change^34^,^35^,^36^,^37^. Understanding how invertebrate assemblages change with climate is therefore important in understanding likely future changes in diversity and the processes driven by diversity. Examination of multi-taxon invertebrate assemblages along elevational gradients allows us to observe the current distributions of different species, make predictions about how they will respond to climate change based on their current climatic envelopes, and identify species that can be used to monitor future range shifts^38^,^39^.

Lepidoptera are ideal for use in climate monitoring, alone or as part of a multi-taxon monitoring tool kit, because they are sensitive to environmental variables and their herbivorous life histories tie them closely to larger community-level shifts^40^,^41^. They are easy to sample in large numbers using automated light traps, giving strong statistical power, and they are relatively well known taxonomically^42^. Lepidoptera have been used extensively as indicators elsewhere, in studies of vegetation health, restoration and fragmentation^37^,^43^,^44^,^45^,^46^, and in studies of change along latitudinal and elevational gradients^47^,^48^.

We examined elevational distributions of canopy and understorey moth species along three forested, elevational transects, which encompass different climatic bioregions within Yunnan Province, south-west China (Fig. 1). The sub-alpine Lijiang transect lies within, and the sub-tropical Ailao Shan transect is immediately adjacent to the South Central China biodiversity ‘hotspot’ , while the tropical Mengla transect is situated in the small area of Chinese territory which Mittermeier *et al*.^4^ places in the Indo-Burma ‘hotspot’. We predict that if tropical species are more sensitive to changes in climatic conditions than temperate species, the assemblage composition of tropical Mengla will be more strongly elevationally stratified and have a larger number of species that are elevationally restricted, compared with the two other, more temperate, elevational transects.

## Results

### Species richness across latitude and elevation

Across all three elevational transects, a total of 48358 individual moths were sampled. At Mengla (tropical rain forest) we sampled a total of 13213 individuals, belonging to 2160 morphospecies. At Ailao Shan (sub-tropical evergreen broad-leaved forest) we collected 19952 moths belonging to 1438 morphospecies. At Lijiang (sub-alpine coniferous forest), we collected 15193 individuals, belonging to 669 species. The proportion of rare species with less than 10 individuals was greatest at Mengla (87%), followed by Ailao Shan (78%) and Lijiang (70%).

Standardised species richness clearly differed among the three transects, and was highest in tropical Mengla followed by sub-tropical Ailao Shan and sub-alpine Lijiang (Fig. 2). Within each of the three transects, however, species richness did not show consistent patterns in response to elevational differences. This was statistically confirmed by the results of the generalised linear mixed effect model which showed an insignificant effect of elevation (F = 0.02, P = 0.88) after controlling for the differences among the three transects.

### Elevational stratification of moths

Regardless of the similarity index used, species assemblages exhibited high levels of elevational stratification along all three gradients (Fig. 3, Supplementary Fig. S2). This suggests that the clear elevational stratification found in this study was not an artefact due to insufficient or different sampling intensities among the survey plots (Chao-Sørensen estimated similarity values, Fig. 3) or differences in species richness (Raup-Crick similarity values, Supplementary Fig. S2). The results of PERMANOVA confirmed statistically that elevation was a significant factor for all three transects regardless of similarity index used (P < 0.001 for all three transects) with *post hoc* analysis showing significant differences for all pairwise comparisons at P = 0.01 or less.

### Elevation-decay relationships

Elevation-decay relationships showed that assemblage similarity declined linearly with increasing difference in elevation between pairs of plots within each transect (Fig. 4). These relationships were linear for all three elevational transects and similarity indices (except for the Raup-Crick index – see below). For both Sørensen and Chao-Sørensen estimated similarity values, the slope of the decay relationship was steepest at sub-alpine Lijiang, followed by sub-tropical Ailao Shan and tropical Mengla. The same pattern was found for Bray-Curtis similarity values (Supplementary Fig. S3). Although a logistic function trend was seen for Raup-Crick values, no regression analysis was carried out as this index is a non-metric probabilistic measure (Supplementary Fig. S3).

### Indicator species

At the Mengla, Ailao Shan and Lijiang transects, we calculated indicator values of 212, 269 and 126 ‘common’ moth species (see Methods) and, among these, identified 109, 201 and 102 significant indicators, respectively. At Mengla and Ailao Shan, smaller proportions of indicator species were found at the lowest elevations (800 and 2000 m a.s.l. from Mengla and Ailao Shan, respectively), whereas a smaller proportion of indicators was found at the highest elevation at Lijiang (3800 m a.s.l.) (Fig. 5). Across the three transects, the observed numbers of indicator species was substantially greater than any numbers of indicator species generated from 999 null models, suggesting that the observed numbers of indicator species was significantly greater than the number of indicators that would be found by chance (Fig. 6). The highest effect size was observed in Ailao Shan where the largest number of indicator species was also found. Despite the smallest number of indicator species being found in Lijiang, the effect size was greater than that of Mengla (Fig. 6).

## Discussion

As expected, we found the highest number of moth species in the tropical site, followed by the sub-tropical and temperate locations. Within each transect, however, there was no clear pattern of richness with elevation, or mid-elevation peaks. This may be linked to the short elevational span (approximately 600 m) within each elevational transect (but we were unable to find more elevational bands in which to sample due to the presence of human disturbance at lower elevations). However, this is unlikely to fully explain the lack of elevational patterns as we found that the richness of a different group of insects (viz. ants, unpublished data), which were sampled from the same survey plots, showed clear elevational signals. The sheer hyperdiversity of species in the region and associated under-sampling may also play a role. We encountered much higher species richness in Yunnan compared with that from tropical and sub-tropical Australian rainforests^49^ using identical sampling protocols (e.g. 2160 species in Mengla compared to 1134 species in a north Queensland tropical rainforest). As noted in the Introduction, the very high species richness we observed in south-western China may reflect a number of coincident factors. Yunnan is located in a land-locked area of the Eurasian supercontinent and, following the principles of island biogeography, should have a very large source fauna. It is also a point of overlap between biogeographic regions (the Palaearctic and Oriental in Wallacean terms). These factors, in combination with the highly heterogeneous mountainous landscape and the associated ecosystem (and environmental) variability across a wide range of elevations and latitudes all will contribute to the very great biological diversity of the region. Yunnan, indeed, is by far the most biodiverse of all Chinese provinces^50^.

The highly diverse forest ecosystems in Yunnan are under threat from a range of intercorrelated environmental pressures, including habitat loss and fragmentation, driven by a large and burgeoning human population^50^,^51^. In tandem with many other environmental impacts, climate change has already had significant effects on the region^52^, and is predicted to have future impacts in line with global predictions^52^,^53^. In order to monitor the impacts of climate change in this biodiversity hotspot through time, we suggest the use of a ‘predictor set’^12^,^39^ of elevationally restricted species. The wide range of formally defined indicator species we have identified across all three forest types and all the sampled elevations within them guarantees the effectiveness of using such an approach to monitor future change overcoming any localised impacts through habitat change – which would be a challenge if only a small number of ‘indicators’ were available. We, however, found more than 100 indicator species in each of our elevational transects spread across different elevational bands (Fig. 5). Some of these species have been barcoded (DNA barcode data deposited in BOLD systems, boldsystems.org) and will be included in predictor sets for future monitoring. We have established groups of permanent, replicated elevational plots which can be monitored through time. The vegetation assemblages at each site have been surveyed on these plots, and a suite of environmental variables (temperature, precipitation, soil properties) are being monitored.

We have shown that an elevational change of just 200 m drives significant change in the assemblage composition of moths in each of the three forest types studied. This result is in agreement with similar studies using exactly comparable methodology that we have carried out in Australian rain forests^49^. The fact that these substantial and significant changes in community structure occur over a mere 200 m vertical interval, and in such a relatively mobile taxon as the moths, has considerable implications for conservation under scenarios of climate change. A 200 m vertical change at any one latitude represents about 1.5 °C change in average temperature – less than is predicted for global change over the next half century or so^54^.

The ecological impacts of climate change are predicted to be more severe in the tropics compared with the temperate zone, as tropical species evolved in a more stable environment (diurnally, seasonally and/or across years in recent historical time scales)^25^,^55^,^56^,^57^,^58^ potentially leading to more severe distribution shifts, phenological mismatches or extinction^26^,^27^,^59^,^60^. Despite initial expectations, the present study found that temperate and sub-tropical moth assemblages were actually more sensitive to climatic gradients than the tropical assemblages. This result, however, need to be interpreted with care as the impacts of elevation and latitude may be confounded in our study due to the nature of Yunnan’s topography whereby higher elevations are found in higher latitudes. Our three transects, unavoidably, sampled different absolute ranges in each case.

The observed higher sensitivities of moths in Ailao Shan and Lijiang compared with those of Mengla were demonstrated by both the number of elevationally restricted species (using effect size to control for differences in gamma diversity across elevational transects) (Fig. 6) and the degree of beta turnover (i.e. rate of species turnover across elevations) (Fig. 4). Previous studies of tropical versus temperate climate sensitivity have been based on laboratory measurements, modelling or vertebrate data, which can be difficult to generalize to real-world responses or less mobile organisms^61^,^62^. For example, Freeman *et al*.^62^ found that tropical bird species are moving upslope faster than temperate birds, suggesting higher tropical climatic sensitivity. Rehm *et al*.^63^ analysed plant and animal distributions separately and found differences in range shifts between these groups, concluding that climate-driven distribution shifts may also be related to dispersal abilities and life history traits.

Apart from temperature, a suite of other environmental factors shift across elevation, including solar radiation, wind speed and available host plants. We encountered the fewest elevationally restricted species at the highest elevations of our sub-alpine location, where harsh conditions may limit the number of restricted, resident, species (Fig. 5). With further climate change, sub-alpine species may readily track their climatic envelopes to higher elevations. This, however, is only possible if there is higher elevation habitat available at which appropriate floristic changes have occurred. Resident moth species, as herbivores, depend on the presence of appropriate larval host plants. If plants are slower to respond and track climatic envelopes^63^, we may see a mismatch in the upward movement of herbivores and their host plants. Low elevation species in the temperate zone have been shown to expand their ranges to include to higher elevations^64^. As our sub-alpine transect occurs in a region where much of the lower elevation forest has been cleared, we predict there will be a gap between climate-driven shifts of low elevation species moving upwards and an absence of species available to move in to the low elevation habitat, a form of biotic attrition^31^ that may result in overall species loss in this area.

Climate change is already having a marked impact on the elevational and latitudinal distributions of Lepidoptera. Most of the demonstrated distribution shifts, however, are from temperate areas in Europe and the USA, where long-term data sets are available^65^,^66^,^67^,^68^,^69^. One exception is a historical data set of moths from Mt Kinabalu, Borneo, that allowed Chen *et al*.^70^ to resample and document an average upslope shift of 67 m in geometrid moth assemblages between 1965 and 2007. These upslope shifts in moth assemblages may be a direct response to changes in climate, a response to shifts in host plants, or a combination of physiological responses and community level interactions. The present study provides valuable baseline data for a biodiversity hotspot that has been little examined in terms of arthropod assemblages, which will allow for future monitoring of distribution shifts.

## Methods

### Study area and general procedures

**Figure 1:**
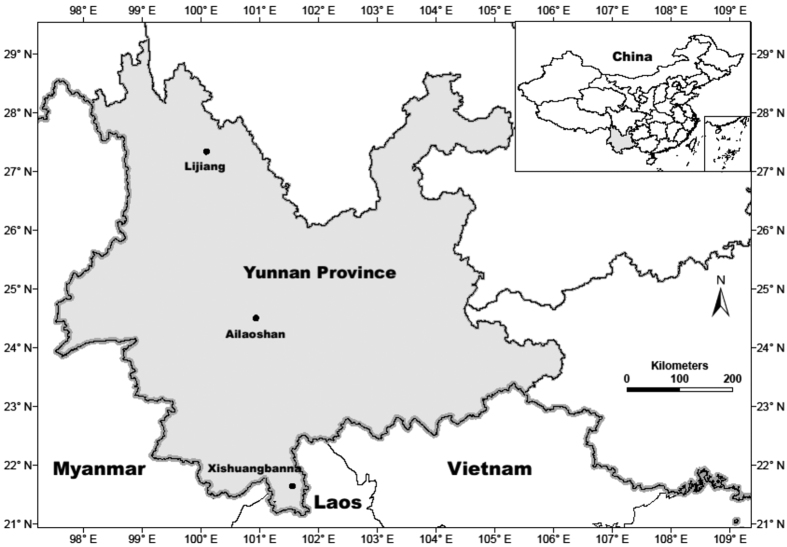
Map showing the locations of the study transects. Elevational transects were located in tropical Xishuangbanna (Mengla), sub-tropical (Ailao Shan) and sub-alpine (Lijiang) Yunnan Province, China. The map was generated using ArcGIS 10.1 (www.esri.com).

**Figure 2:**
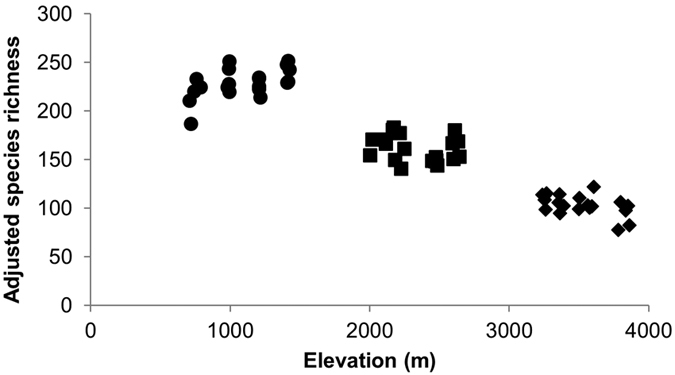
Species richness plotted against the elevations of the survey plots. Species richness was adjusted to an abundance of N = 364 (see Methods for more details) and actual elevations (not elevational bands) were used (⦁, Mengla; ◾, Ailao Shan;◆, Lijiang).

**Figure 3:**
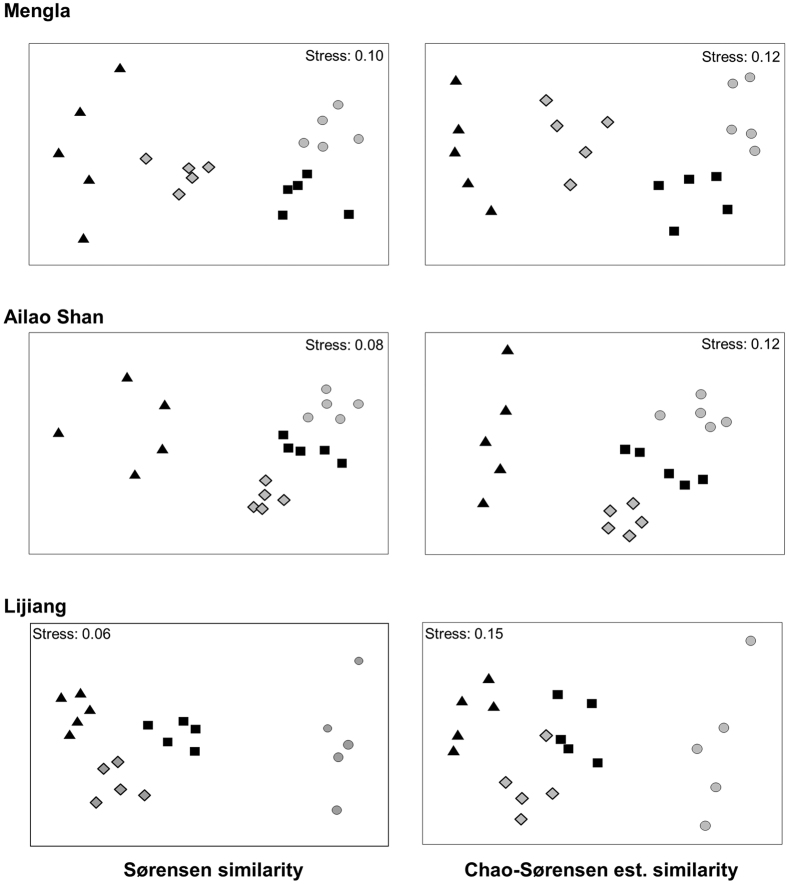
NMDS ordinations based on Sørensen (left) and Chao-Sørensen estimated (right) similarity matrices of moth assemblages across the three elevational transects. Different elevational bands are represented by the following symbols: for Mengla, ▲ = 800 m, 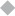 = 1000 m, ■ = 1200 m, 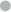 = 1400 m; for Ailao Shan, ▲ = 2000 m, 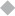 = 2200 m, ■ = 2400 m, 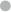 = 2600 m; and for Lijiang, ▲ = 3200 m, 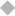 = 3400 m, ■ = 3600 m, 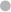 = 3800 m.

**Figure 4:**
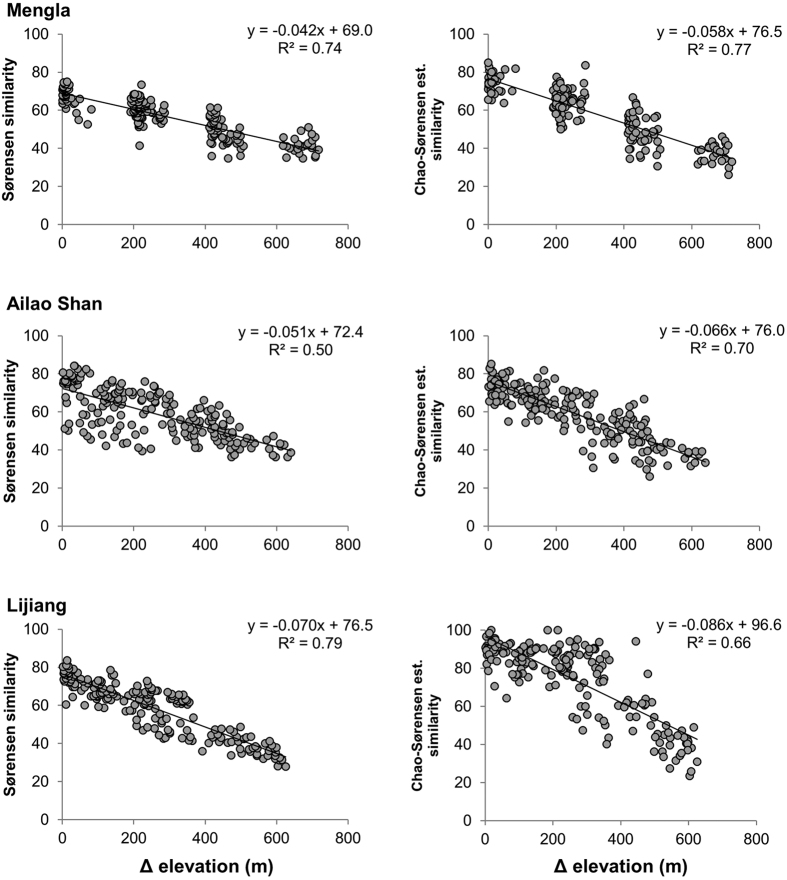
Elevation-decay relationships of moth assemblages. Similarity values of moth assemblages were plotted against pair-wise differences in elevation (Δ elevation) of the survey plots. Similarity values are based on Sørensen (left) and Chao-Sørensen estimated (right) similarity values. Straight trend lines were drawn with regression coefficients and R^2^ values.

**Figure 5:**
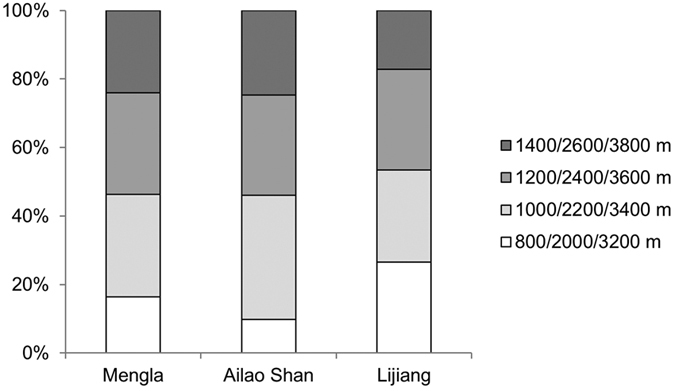
Proportion of the number of significant indicator species indicative of different elevational bands.

**Figure 6:**
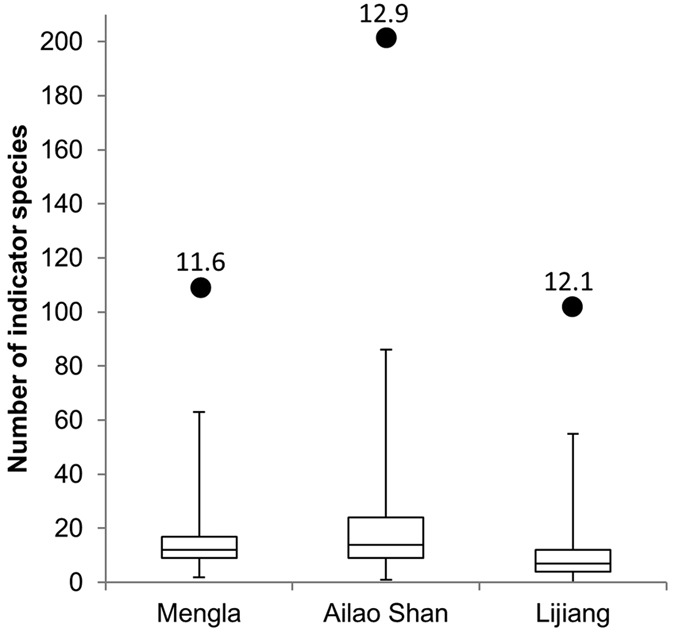
Number of indicator species observed from actual data and null models. The numbers of observed moth indicator species were shown as black dots, and those derived from 999 permuted null datasets were shown as box plots from the first to third quartiles with whiskers from maximum to minimum. Effect size (see Methods) is shown above the observed number of indicator species.

Three permanently marked elevational transects were established in Yunnan Province, south-west China in tropical (Mengla) (22°N), sub-tropical (Ailao Shan) (24°N) and temperate (Lijiang) (27°N) forests, each about 300 km apart along a south-north bearing (Fig. 1). Twenty 20 × 20 m sampling plots were established on each transect, grouped into sets of five plots at each of four elevational bands separated by vertical intervals of approximately 200 m. Locations of the sampling plots were chosen carefully so as to avoid any visible disturbances (e.g. canopy gaps) and to maintain similar aspects and slope where possible. The elevational range sampled at each transect varied; 800–1400 m a.s.l. at Mengla, 2000–2600 m a.s.l. at Ailao Shan and 3200–3800 m a.s.l. at Lijiang. Extensive botanical and insect surveys have been carried out at each sampling location. Moths were sampled using Pennsylvania light traps^71^ modified for use in forests (with a basal battery holder and an upper rain cover). The same protocols have been used to sample moths along Australian elevational transects^49^ and at other locations globally^72^. At each plot a trap was situated at ground level and another in the canopy. At Mengla and Ailao Shan, ground and canopy traps were run for three nights, producing a total of 120 samples from each transect. Due to unavoidable time constraints, at Lijiang some plots were sampled for only two nights, or until we encountered at least 100 individuals (either 2 or 3 nights) in each layer (canopy and ground), producing a total of 98 samples. All samples were processed on site with macromoths (wing length greater than 1 cm) and all Pyraloidea extracted, identified to family and morphospecies, and counted. Voucher collections of pinned, set specimens were established for each transect and have been deposited in the Kunming Institute of Zoology, China.

### Mengla (Latitude ca 21.5°N).

The tropical rain forest elevational transect was located near the village of Bubeng, Mengla, in the Xishuangbanna Prefecture, close to the international border with Laos. The area’s climate is monsoonal, with a pronounced wet season between May and October and a dry season between November and April. The average annual temperature at Mengla is 21 °C, with an average annual rainfall of 1530 mm, of which only 281 mm falls during the dry season. Five plots in each of four elevational bands (800, 1000, 1200 and 1400 m above sea level (a.s.l.)) were established. Plots at 800 m a.s.l. were dominated by *Parashorea chinensis* and *Pittosporopsis kerrii*; those at 1000 m a.s.l. by *Aporusa yunnanensis* and *Pittosporopsis kerrii*; those at 1200 m a.s.l. by *Castanopsis echinocarpa* and *Lithocarpus truncatus*; and those at 1400 m a.s.l. by *Castanopsis mekongensis* and *Lithocarpus truncatus.* Moths were sampled between the 5^th^ and 24^th^ of July 2012.

### Ailao Shan (Latitude ca 24.5°N).

Located within the larger Ailao Mountains Reserve, the sub-tropical elevational transect was in the Qian Jia Zai area, about 200 km south-west of Kunming. The Ailao Mountains Reserve is a protected forest covering 504 km^2^. The sub-tropical climate of the Qian Jia Zai area has an average temperature of 11 °C and average annual rainfall around 1900 mm. The Ailao Mountains occur at a major climatic border between the south-west and south-east monsoon systems of China^73^.

At Ailao Shan, five plots were located in each of four elevational bands; 2000, 2200, 2400 and 2600 m a.s.l. The lowest plots (2000 m a.s.l.) were dominated by *Claoxylon khasianu, Manglietia insignis* and *Lithocarpus truncatus;* plots at 2200 m a.s.l. by *Lithocarpus hancei, Camellia sinensis* and *Cyclobalanopsis stewardiana*; those at 2400 m a.s.l. by *Rhododendron leptothrium, Lithocarpus xylocarpus, Eurya obliquifolia* and *Eura paratetragonoclada*; and, the highest plots at 2600 m a.s.l. by *Castanopsis wattii* and *Rhododendron leptothrium.* Bamboo was a large component of the understory at 2000 m a.s.l., but declined with increasing elevation. Moths were sampled between the 1^st^ and 20^th^ of July 2011.

### Lijiang (Latitude 27.0°N)

The Lijiang transect was located on Yulong Snow Mountain, a massif consisting of thirteen peaks, the highest of which, Shanzidou, reaches 5596 m a.s.l.^74^. This area is a transitional zone between the south-east Tibetan plateau and the north-east Yunnan Plateau. The Lijiang region has experienced recent climate warming, mean temperatures between 1999 and 2008 were 1.17 °C higher than those between 1979 and1988^75^, and precipitation has generally increased since the 1980’s^76^. This region is also under substantial environmental pressures from steadily increasing tourist visitation^75^. Precipitation in Lijiang is highest from May to October, and averages 968 mm annually while the average annual temperature is 12.6 °C^76^.

Five replicate plots were established at each of 3200 m, 3400 m, 3600 m and 3800 m a.s.l. Dominant plant species were *Abies forrestii* and *Quercus pannosa* at 3200 m a.s.l.; *Abies georgei* and *Quercus pannosa* at 3400 m a.s.l. and *Abies georgei* at both the 3600 m and 3800 m a.s.l. elevational bands. Moths were sampled at Lijiang between the 9^th^ and 22^nd^ of August 2012.

### Analysis

Although vertical stratification of moth assemblages was evident, many species were shared between the canopy and understorey within a plot^72^. We therefore pooled canopy and understorey moth catches at each plot and treated them as a single sample.

We first compared moth species richness at different elevational bands across the three transects. The number of species collected from each plot, however, varied greatly (Supplementary Fig. S1) and was positively correlated with the number of specimens collected (correlation coefficient = 0.43). Consequently, species richness was adjusted to the smallest sample size of N = 364 individuals (i.e. smallest number of individuals collected among the 60 survey plots), using abundance-based species accumulation curves generated from EstimateS ver. 9.1.0^77^. Using the *lme4* package^78^ (ver 1.1–10) available in R^79^ (ver. 3.2.2) we ran a linear mixed effect model with transect as a random factor and elevation as a predictor. The P value was calculated based on the Kenward-Roger approximation available from the *afex* R package^80^.

Variations in moth assemblage composition were investigated using four complementary similarity indices: Sørensen, Bray-Curtis, Chao’s abundance-based Sørensen (‘Chao-Sørensen estimated’) and Raup-Crick. For the first two indices, only ‘common’ species were included to calculate their values, whereas all species were used for the latter two indices. Common species were selected following Novotny *et al*.^81^ who calculated the probability of observing a species (P) given N individuals from n plots, under an assumption of the extreme case where there is no beta-diversity within a given habitat, using the following equation: P = 1 − (1 − 1/n)^N^. Based on this equation, we calculated that the minimum threshold abundance of ‘common’ species was 14: that is, this suggests a P=95% chance of detection from n = 5 plots within each elevational band. Abundances of ‘common’ species were then transformed to presence/absence binary data to calculate values of the Sørensen similarity index for pairs of plots. Proportional abundances of ‘common’ moth species (relative to the total number of individuals of all moth species collected within each plot) were incorporated using the Bray-Curtis similarity index. Unlike the Sørensen and Bray-Curtis indices, the Chao-Sørensen estimated index (calculated using EstimateS software) estimates the extent of shared species between two plots, based on the number of observed rare, shared species. The Chao-Sørensen index is effective when samples contain a substantial proportion of rare species due to undersampling^82^. The Raup-Crick index is a probabilistic resemblance measure (calculated using the *vegan* community ecology package available within R)^83^ which returns the probability that two observed plots will have a greater number of shared species than two plots with the same number of species which are randomly drawn from the species pool of a given habitat. The Raup-Crick index therefore measures beta-diversity while controlling for sampling effects caused by differences in alpha-diversity^84^.

Using these four similarity indices, we generated non-metric multi-dimensional scaling (NMDS) ordinations to assess visually patterns of moth species composition across different elevational bands within each of the three transects. All NMDS ordinations were generated using PRIMER6 software^85^ with 25 random restarts and the first Kruskal fit scheme. We tested the differences in moth assemblage composition among elevational bands, using permutational multivariate analysis of variance (PERMANOVA) implemented in PRIMER6 and PERMANOVA+ add-on software^86^. As almost all moth species were unique to each elevational transect, similarity values were very low or zero between plots from different elevational transects. For this reason we carried out three separate PERMANOVAs for the three transects. Pseudo-F statistics were calculated using Type III sums of squares, and P values using 4999 unrestricted permutations.

To quantify the sensitivity of moths to elevational differences across the three transects, we compared the strength of the elevation-decay relationship (decreasing assemblage similarity with increasing elevational differences) and the number of species restricted to a certain range of elevations. To investigate elevation-decay relationships, we plotted similarity values of moth assemblages against inter-plot differences in elevation. For each transect, four different plots were generated using the four similarity indices mentioned above. Trend lines were fitted on the plots to calculate slopes, intercepts and r-square values. The slope of the fitted line should be steeper if moth assemblages are more sensitive to changes in elevation.

To identify species with elevationally restricted distributions, we adopted the indicator value protocol developed by Dufrêne and Legendre^87^. The indicator value protocol assesses the ‘value’ of species as indicators of certain elevational bands by quantifying the species specificity and fidelity, expressed as a percentage. A maximum indicator value of 100% is given to a species if it occurs exclusively within certain elevational bands (maximum specificity) and at all sampling plots within these elevational bands (maximum fidelity). We calculated the indicator values of all species for each individual elevational band (e.g. 800 m, 1000 m at Mengla) and all possible ranges of sequential elevations (e.g. 800–1000 m, 800–1200 m, 1200–1400 m), excluding only the entire range of the transect (800–1400 m), giving a total of nine indicator values per species within each transect. A species was deemed indicative of the elevational band or range where the highest indicator value was attained for that species. The significance of this indicator value was then tested by permuting the samples 999 times. We then summed the number of significant indicator species for each elevational transect. The total number of significant indicator species, however, cannot be compared directly because the total number of species varied greatly among transects. We therefore adopted the method developed by Nakamura *et al*.^38^ who quantified the departure of the number of indicator species from those based on the null model. To this end, we generated a total of 999 permuted datasets by shuffling the samples. We then quantified the departure (i.e. the effect size) for each of the three transects by calculating the difference in the number of significant indicator species between the observed and the mean of the null datasets divided by the standard deviation of the null datasets.

## Additional Information

**How to cite this article**: Ashton, L. A. *et al*. Elevational sensitivity in an Asian ‘hotspot’: moth diversity across elevational gradients in tropical, sub-tropical and sub-alpine China. *Sci. Rep.* **6**, 26513; doi: 10.1038/srep26513 (2016).

## Supplementary Material

Supplementary Information
