# Supplementary material for: Elevational sensitivity in an Asian ‘hotspot’: moth diversity across elevational gradients in tropical, sub-tropical and sub-alpine China
Source: Sci Rep. 2016 May 23;6:26513. doi: 10.1038/srep26513 (PMC4876391; doi:10.1038/srep26513)
Supplement: Supplementary Information [file srep26513-s1.pdf]

Scientific Reports

SUPPLEMENTARY INFORMATION

Elevational sensitivity in an Asian 'hotspot': moth diversity across elevational gradients in tropical, sub-tropical and sub-alpine China

Louise A. Ashton, Akihiro Nakamura, Chris J. Burwell, Yong Tang, Min Cao, Terry Whitaker, Zhenhua Sun, Hua Huang and Roger L. Kitching

Supplementary Fig. S1 Observed moth species richness and abundances plotted against the actual elevations of the survey plots.

Supplementary Fig. S2 NMDS ordinations based on Bray-Curtis and Raup-Crick similarity matrices of moth assemblages.

Supplementary Fig. S3 Elevation-decay relationships based on Bray-Curtis and Raup-Crick similarity values

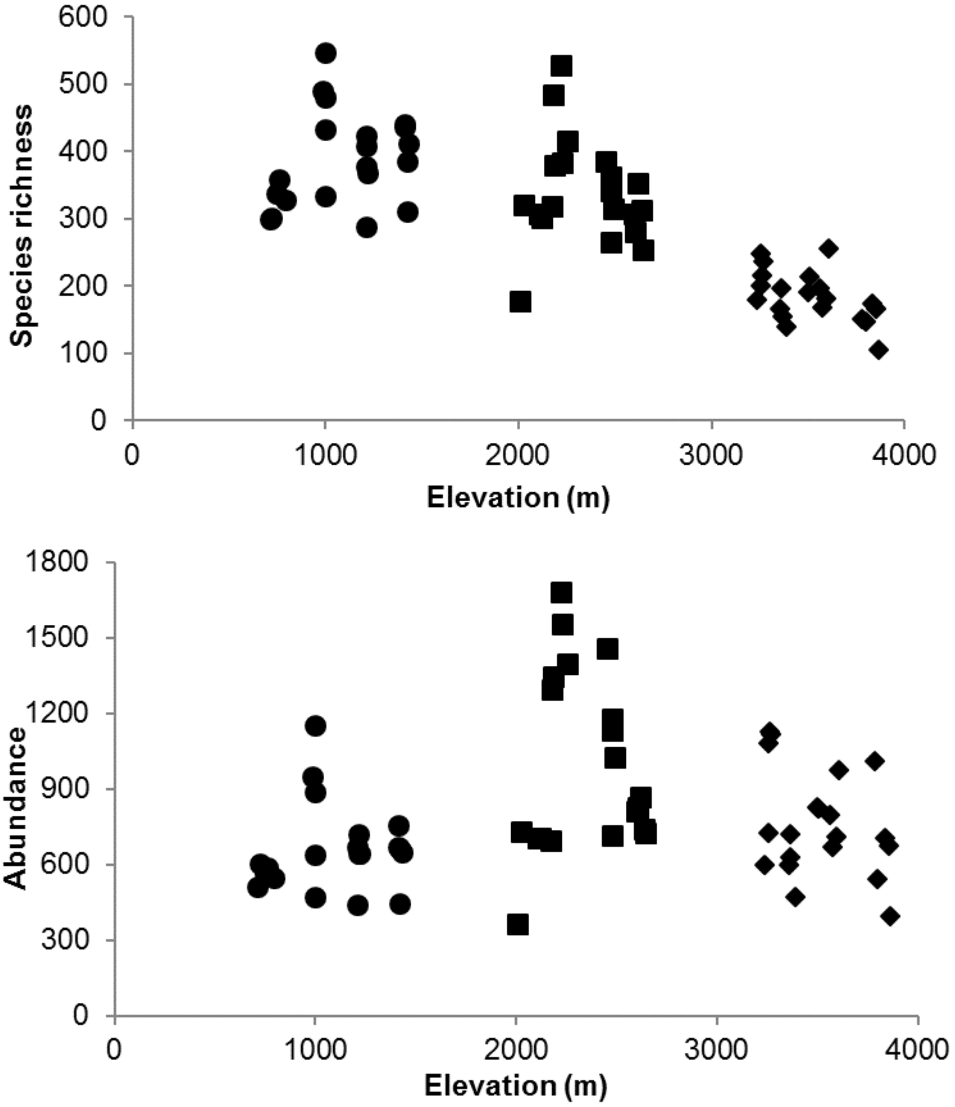

Supplementary Fig. S1 Observed moth species richness and abundances plotted against the actual elevations of the survey plots across the three transects (●, Mengla; ■, Ailao Shan; ◆, Lijiang).

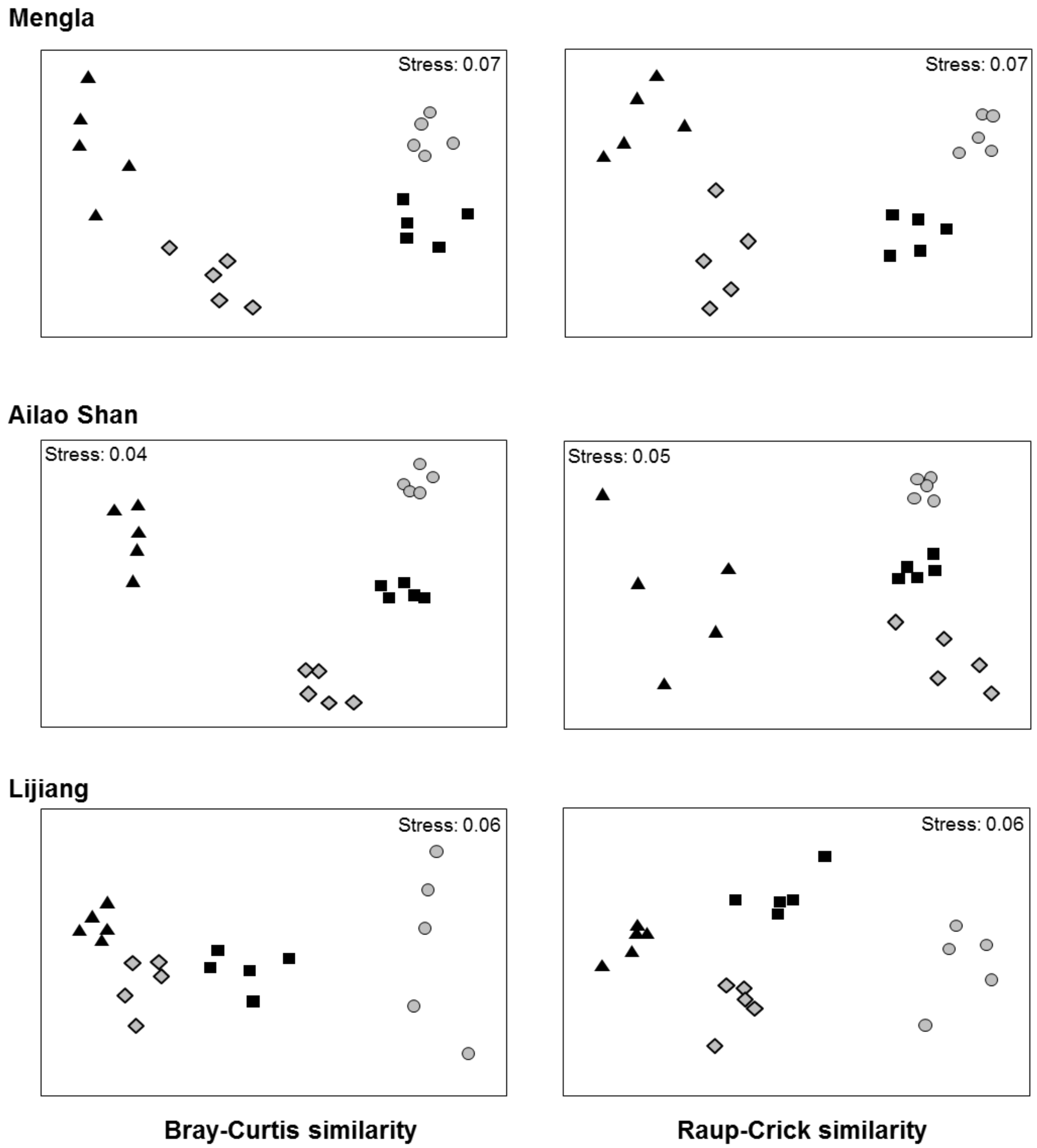

Supplementary Fig. S2 NMDS ordinations based on Bray-Curtis (left) and Raup-Crick (right) similarity matrices of moth assemblages across the three elevational transects. Different elevational zones are represented by the following symbols: for Mengla, ▲ = 800m, ◆ = 1000m, ■ = 1200m, ● = 1400m; for Ailao Mt., ▲ = 2000m, ◆ = 2200m, ■ = 2400m, ● = 2600m; and for Lijiang, ▲ = 3200m, ◆ = 3400m, ■ = 3600m, ● = 3800m.

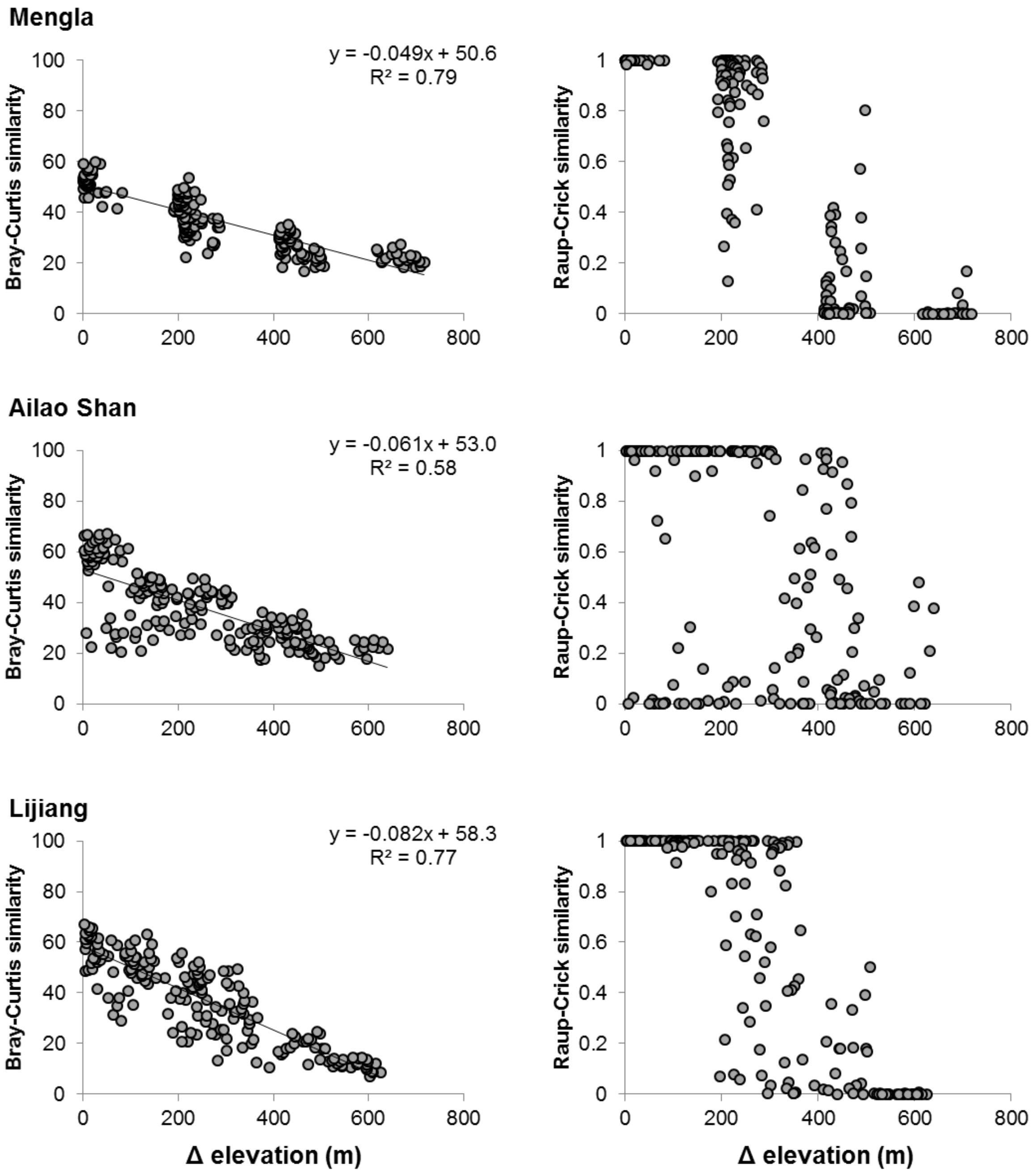

Supplementary Fig. S3 Elevation-decay relationships showing similarity values plotted against pair-wise differences in elevation (Δ elevation) of the survey plots within each elevational transect. Similarity values are based on Bray-Curtis (left, using only common moth species at N > 14 individuals) and Raup-Crick (right, using abundance data of all moth species) similarity values. Straight trend lines were drawn for Bray-Curtis similarity values with regression coefficients and R^2 values. Trend lines were not drawn for Raup-Crick similarity values as these values are non-metric.
